# Untargeted metabolomic dataset of leaves from twenty-four accessions of wild and cultivated tomato plants

**DOI:** 10.1016/j.dib.2026.112828

**Published:** 2026-05-07

**Authors:** Komla Exonam Amegan, Florent Magot, Alan Kergunteuil, Bernard Caromel, Anne-Violette Lavoir, Romain Larbat

**Affiliations:** a INRAE, Université de Lorraine, LAE, INRAE, F-54000 Nancy, France; b INRAE, GAFL, CS 60094, F-84140 Montfavet, France; c Université Côte D’Azur, INRAE, UMR ISA, 06000 Nice, France; d Université de Tours, Biomolécules et Biotechnologies Végétales, EA2106, F-37200 Tours, France; e INRAE, PSH, F-84000 Avignon, France; f Institut Agro, Université d’Angers, INRAE, IRHS, SFR QUASAV, F-49000 Angers, France

**Keywords:** Metabolite, Solanaceae, Interspecific and intraspecific diversity

## Abstract

Tomato (*Solanum lycopersicum* var. *lycopersicum*), one of the most important crops worldwide, has a complex domestication history that began in Latin America, region hosting also fourteen wild relative species and subspecies. Domestication and subsequent breeding efforts have led to the development of the modern cultivated tomato, prized for its agronomic performance and economic value. However, this process also resulted in a substantial erosion of genetic and metabolic diversity, potentially limiting the plant’s adaptive capacity and resilience to environmental stresses.

Previous comparative studies between domesticated tomato cultivars and wild relative species have underscored the evolutionary shifts in various plant traits associated with biotic stress. Yet, most of these studies relied on a limited number of wild accessions, reflecting a general tendency to underestimate their genetic and metabolic diversity.

In this study, we sought to characterize both intra- and inter-specific metabolic diversity in tomato and its wild relatives. Using Liquid Chromatography High Resolution Mass Spectrometry (LC-HRMS) analysis, we profiled the chemical composition of hydro-methanolic leaf extracts from twenty-four accessions representing five *Solanum* species and subspecies, each with distinct natural histories and domestication levels. This dataset provides a comprehensive overview of leaf metabolic diversity across cultivated and wild tomato species, offering insights into the evolutionary and ecological forces shaping specialized metabolism within the tomato clade. It is available at https://doi.org/10.57745/QM0BOR*.*

Specifications Table

**Table:** 

Subject	Biology
Specific subject area	*Agricultural Sciences*
Type of data	Raw and processed data files as [.mzXML] and [.mgf] and table as [.xls]
Data collection	Data were collected from cultivated and wild tomato accessions provided by the Biological Resources Centre “Leg” (CRB-Leg, INRAE Avignon; https://eng-gafl.paca.hub.inrae.fr/vegetable-germplasm-centre/crb-leg-collections). Seedlings were grown in small cubic pots filled with a mixture of potting soil. They were placed in a climatic chamber under the following conditions: 24 ± 1 °C, 60–70% relative humidity, 16:8 h light: dark photoperiod. Two fresh leaflets from 8–10 seedling plants (4- to 6-week-old) of each accession were harvested to prepare hydro-methanolic extracts. Liquid Chromatography High Resolution Mass Spectrometry (LC-HRMS) analyses were acquired on an Orbitrap IDX™ (ThermoFisher Scientific, Bremen, Germany) mass spectrometer in positive and negative electrospray ionization modes.
Data source location	Data collected in the Lorraine University facilities (PEPLor and PASM platforms)
Data accessibility	Repository name: Recherche Data Gouv repository https://recherche.data.gouv.fr/ Data identification number: 10.57745/QM0BOR Direct URL to data: https://doi.org/10.57745/QM0BOR Instructions for accessing these data: Licenses of use: etalab 2.0 (https://spdx.org/licenses/etalab-2.0.html)
Related research article	*None*

## 1 Value of the Data

• This dataset allows characterizing the intra- and inter-specific leaf metabolic diversity on cultivated and wild tomato plants.
• This metabolomics dataset can be used to map the distribution of metabolic classes across *Solanum* species
• Combined with dataset covering phenotypic traits of the analysed genotypes, this dataset can help identifying correlated or putative causal metabolites for the traits.

## 2 Background

In this study, we aimed to characterize both intra- and inter-specific metabolic diversity in tomato and its wild relatives. The selected species collectively represent a large part of the ecological variation found among the fourteen species and subspecies related to domesticated tomato (See [1] for phylogenetic trees). From a geographical viewpoint, *Solanum cheesmaniae* is endemic to the Galapagos Islands, whereas *Solanum habrochaites* and *Solanum pennellii* are differentially distributed across the Andes, from Ecuador to Peru and Peru to Chile, respectively [2]. More generally, Ramírez-Ojeda et al [3], demonstrated that these species belong to distinct ecological clusters characterized by diverse ecogeographic, climatic, and edaphic conditions, and display variable levels of intraspecific diversity. In our study, for each species, we integrated several accessions that can be considered as population reflecting intra-specific diversity. Using Liquid Chromatography High Resolution Mass Spectrometry (LC-HRMS) untargeted analysis, we profiled the chemical composition of hydro-methanolic leaf extracts from twenty-four accessions representing five *Solanum* species and subspecies.

## 3 Data Description

This dataset contains LC-HRMS data from leaf hydro-methanolic extracts of twenty-four accessions of wild and cultivated tomato plants. The accessions included in this study were selected to represent a broad spectrum of genetic diversity. These comprised 6 *S. lycopersicum* accessions, in which both large-fruited varieties and cherry-type (var. *cerasiforme*) were considered together, as well as wild species such as *S. cheesmaniae* (10)*, S. habrochaites* (6), and *S. pennellii* (2)*.*

**Fig. 1:**
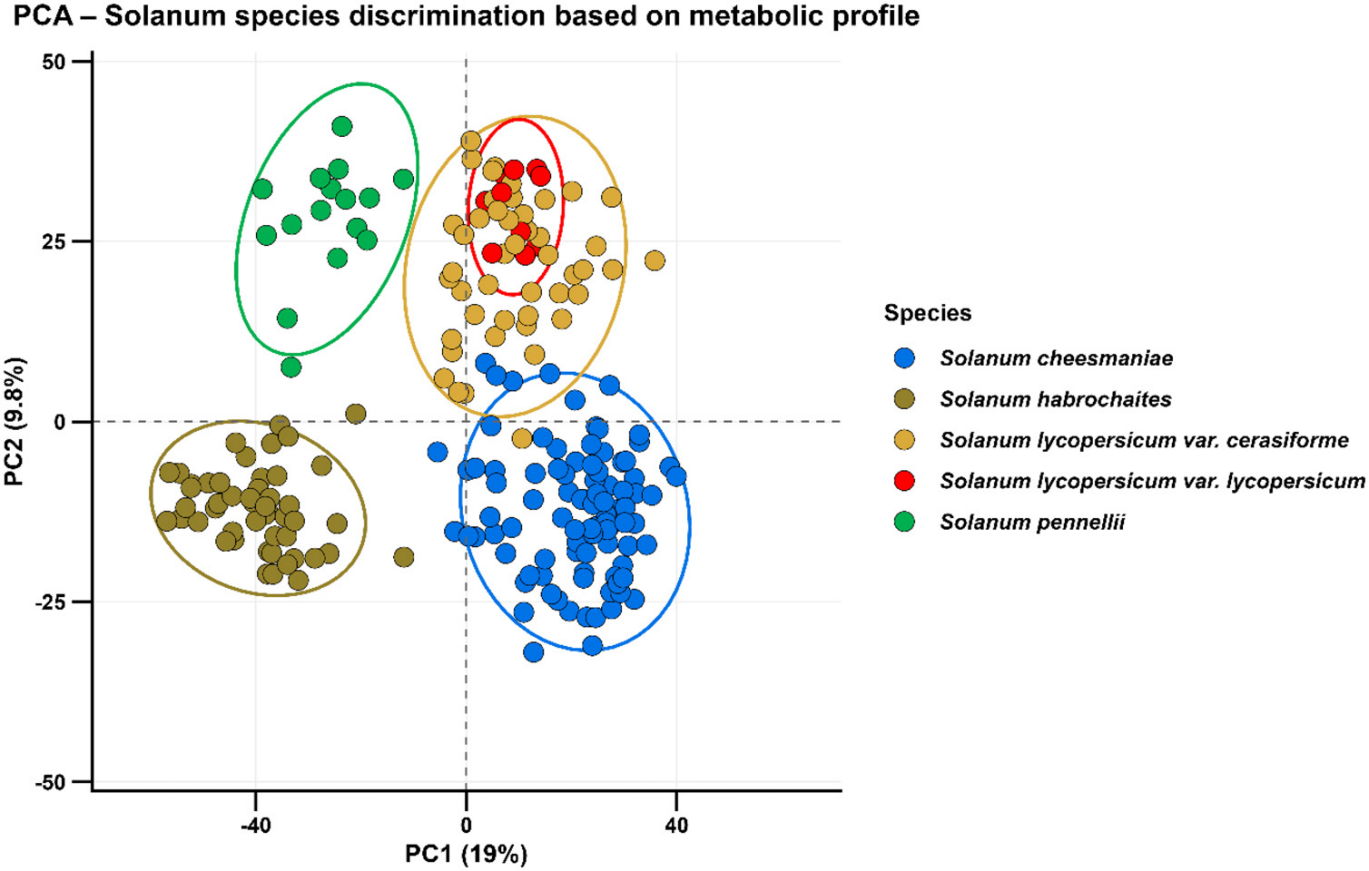
Metabolic profile clustering of *Solanum* species by principal component analysis.

LC-HRMS analyses were acquired on an Orbitrap IDX™ (ThermoFisher Scientific, Bremen, Germany) mass spectrometer in positive and negative electrospray ionization modes (ESI). Each raw file has a code name which correspondence in Solanum species, subspecies and accession is given in the metadata table “Solanum metadata 14-11-25.tab” [4]. MS^1^ raw files were processed using Compound discoverer 3.3 software (ThermoFisher Scientific, Bremen, Germany) with an analytical workflow described in the “Analytic_Workflow_Compound_Discoverer.txt” file [4]. Processed MS^1^ data led to the detection and quantification of 3764 metabolic features across the 217 samples (24 accessions x 9–13 replicates). The MS^1^ quantification on all samples is given as a table (Solanum data 14-11-25.tab, in [4]). Each line represents one metabolic feature, while samples are indicated in columns (from the 7th to the 224th column). The quantitative variable for each metabolic feature corresponds to the surface area under the chromatogram baseline, which is proportional to ionization properties of metabolic features and to their relative abundance in samples. The columns 1 to 6 furnish information on the metabolic features (measured *m/z*, retention time, calculated molecular weight and a proposed molecular formula according to its ionisation mode) together with the availability of MS^2^ data. Additional columns (from the 225th to the 244th) correspond to quantitative values measured for two blank samples and the 18 QC samples injected throughout the whole sequence analysis. MS^2^ analysis was realized on a mixture of all samples which allows to obtain fragmentation data on 3412 features. MS^2^ data is contained in a mascot generic format file (HCD.mgf in [4]). Each MS file has been generated as “.raw” (Thermo format). For accessibility considerations, each MS file has been converted in the generic format (“.mzXML”), with one file containing data acquired in positive mode (POS) and another file containing data acquired in negative mode (NEG). Principal Component Analysis was generated from the quantitative MS^1^ data file. It demonstrates a good data homogeneity across accession replicates together with a strong discrimination power between species (Fig. 1).

## 4 Experimental Design, Materials and Methods

### 4.1 Plant materials and growth conditions

The accessions included in this study were selected to represent a broad spectrum of genetic diversity (for more detail see [1]). These comprised *S. lycopersicum* accessions (6) including large-fruited varieties (var. *lycopersicum)* and cherry-type (var. *cerasiforme*), as well as wild species such as *S. cheesmaniae* (10)*, S. habrochaites* (6), and *S. pennellii* (2)*.*

All the accessions were provided by the Biological Resources Centre “Leg” (CRB-Leg, INRAE Avignon). Seeds were sown in potting soil substrate (Einheitserde EUROHUM’) in small pots (7 × 7 × 8 cm) which were covered with an aluminum foil for three days. *S*eeds from the *S. cheesmaniae* accessions necessitate a 30-min-period of soaking in bleach (24°) in order to speed up the degradation of the outer seed coat and promote germination. The two-week-old seedlings were transplanted in bigger pots (12.8 × 12.8 × 20 cm) filled with the same substrate, and grown in a climatic chamber under the following conditions: 24 ± 1 °C, 60–70% relative humidity, 16:8 h [light:dark] photoperiod. Metabolite extraction was realized on 8 to 10 seedling plants per accessions (4–6-week-old, depending on tomato species and accessions), in order to account growth heterogeneity linked to genetic diversity and to study plants at a vegetative stage with analogous biomass.

### 4.2 Extraction procedure

Two fresh leaflets from 4- to 6-week-old plants of each accession were harvested, immediately frozen in liquid nitrogen and stored at −80 °C until freeze-drying. Then, dry samples were ground into a fine powder and metabolite extraction was conducted following the procedure from [5]. Twenty milligrams of dry powder were extracted with 1 mL of methanol 60%. The mixture was blended for 1 min and then centrifuged for 10 min at 2800 g. In order to increase the final yield, the extraction was repeated once. The two supernatants were pooled and vacuum dried. Then, the residue was dissolved in methanol 70% (200 µL) and filtered (0.22 µm) in vials before MS^1^ analyses. An additional vial was prepared with a pool of each sample (10 µL each) for the MS^2^ analyses.

### 4.3 Untargeted metabolomics analysis

Chromatographic separation was conducted on a Vanquish Ultra-High-Pressure Liquid Chromatography system equipped with a temperature-controlled column XB-C18 Kinetex (150 × 2.1 mm, 2.6 µm) (Phenomenex Inc., Torrance, CA, USA) using a gradient of mobile phase consisting in water + 0.1% formic acid (A) and methanol + 0.1% formic acid (B) at a flow rate of 200 µL·min^−1^. The elution program was composed by a first step from 1% B to 55% B in 14 min, followed by a second step from 55% B to 95% B in 6 min. Then, the column was rinsed for 5 min with 95% B before a 4-min-long re-equilibration step with the initial conditions. The samples (10 µL each) were analyzed randomly.

HRMS^1^ detection was performed on an Orbitrap IDX™ (ThermoFisher Scientific, Bremen, Germany) mass spectrometer in both positive and negative ESI modes. The capillary voltages were fixed to 3.5 kV and 2.5 kV for positive and negative modes, respectively. The source gases were set to 40, 8 and 1 (in arbitrary unit min^−1^) for sheath gas, auxiliary gas and sweep gas, respectively. The vaporizer temperature was 320 °C. Full scan MS^1^ spectra were acquired from 120 to 1200 *m*/*z* under positive ESI mode and from 120 to 500 *m/z* under negative ESI mode, at a resolution of 60,000. The pooled-samples vial was used to performed MS^2^ analysis under data dependent acquisition mode and by applying the AcquireX data acquisition workflow developed by ThermoFisher which increases the number of MS^2^ acquisition on low-intensity ions. The raw data files were processed using the Compound Discoverer 3.3 software to perform the untargeted metabolomics workflow, including peak detection, chromatogram alignment, and feature grouping (parameters provided in the “Analytic_Workflow_Compound_Discoverer.txt” available on the datagouv repository). In addition, the full MS^1^-MS^2^ dataset was exported as a mascot generic format (.mgf) file.

## Limitations

The LC-HRMS analyses were performed on leaf extracts from young plants (4–6 weeks) grown in climatic chambers under non stress conditions. As such, this dataset represents the leaf constitutive composition, excluding metabolites specifically induced under biotic or abiotic stress or accumulated in latter phenologic stage.

This dataset compiles data acquired in both negative and positive ESI mode from 120 to 500 *m/z*, but only in positive ESI mode from 500 to 1200 *m/z.*

## Ethics Statement

The authors have read and followed the ethical requirements for publication in Data in Brief and confirm that the current work does not involve human subjects, animal experiments, or any data collected from social media platforms*.*

## CRediT Author Statement

**Komla Exonam Amegan**: Conceptualization. Data acquisition. Formal Analysis. Writing - review/editing. **Florent Magot**: Methodology. Data acquisition. Data curation. Writing -review/editing. **Alan Kergunteuil**: Data curation. Formal analysis. Writing - review/editing. **Bernard Caromel**: Conceptualization. Data acquisition. Funding acquisition. Supervision. Writing - review/editing. **Anne-Violette Lavoir**: Conceptualization. Funding acquisition. Supervision. Writing - review/editing. **Romain Larbat**: Conceptualization. Data acquisition. Funding acquisition. Supervision. Writing -original draft/ review/editing*.*

## Data Availability

Recherche Data Gouv repository https://recherche.data.gouv.fr/Untargeted metabolomic dataset of leaves from twenty-four accessions of wild and cultivated tomato plants (Original data)
